# Knowledge, attitudes and practice toward refractive errors management among left-behind children of migrant workers

**DOI:** 10.3389/fpubh.2024.1373209

**Published:** 2025-01-21

**Authors:** Shuang Liu, Guang Yang, Qingnan Li, Ruxia Pei, Shaohua Tang

**Affiliations:** Department of Ophthalmology, Beijing Jishuitan Hospital, Capital Medical University, Beijing, China

**Keywords:** refractive errors, Ophthalmology, left-behind children, knowledge, attitudes, practice, cross-sectional study

## Abstract

**Background:**

This study aimed to access knowledge, attitudes and practice (KAP) regarding refractive errors (RE) management among the left-behind children of migrant workers.

**Methods:**

This cross-sectional study was performed by the Ophthalmology Department of Beijing Jishuitan Hospital between July and August, 2023. The KAP scores were assessed using a self-designed questionnaire.

**Results:**

Analysis of 350 questionnaires showed mean KAP scores of 9.21 ± 3.00 (possible range 0–14), 33.23 ± 3.57 (possible range 9–45), and 50.19 ± 5.31 (possible range 14–70), respectively. Pearson’s correlation analysis showed positive correlation was found between knowledge and practice (*r* = 0.286, *p* < 0.001), negative correlation between knowledge and attitude (*r* = −0.150, *p* = 0.005), and positive correlation between attitude and practice (*r* = 0.141, *p* = 0.008). Multivariate logistic regression analysis showed that children’s age (OR = 0.748, 95%CI: 0.632–0.885, *p* = 0.001), duration away from parents (OR = 0.345, 95%CI: 0.172–0.691, *p* = 0.003) and RE in parents (OR = 0.405, 95%CI: 0.218–0.753, *p* = 0.004) were independently associated with knowledge. Relationship to the child other than grandparent (OR = 0.252, 95%CI: 0.064–0.999, *p* = 0.050), as well as child’s gender (female, OR = 1.671, 95%CI: 1.006–2.777, *p* = 0.047) and duration of sleep per day (OR = 8.401, 95%CI: 1.473–47.923, *p* = 0.017) were independently associated with practice. In addition, structural equation modeling also showed positive impact of knowledge on practice (*β* = 1.251, *p* < 0.001).

**Conclusion:**

Left-behind children of migrant workers have mostly sufficient knowledge, positive attitude and proactive practice toward RE management, significantly influenced by child’s age, relationship with the child, and duration of living without parents.

## 1 Introduction

Refractive errors (RE) correspond to the mismatch between axial length and optical power of the eye, leading to the problem in focusing light on the retina (1, 2). Uncorrected RE are the leading cause of visual impairment worldwide and worldwide blindness (3, 4). Global estimates in 2022 indicated that prevalence of uncorrected RE is as high as 2.26–5.85 per 1,000 individuals below the age of 20 years old (5). Recent systematic meta-analysis reports that astigmatism is the most common among RE (14.9%), followed by hyperopia (4.6%) and myopia (11.7%) (6). However, individual reports discuss much higher rates of myopia among school-aged children in China, up to 32.5% or even 75.4% in some populations, such as competitive elementary schools (7, 8). According to the 2020 study, the prevalence of hyperopia and astigmatism is also high among Chinese preschool children, reported as 13.8 and 17.6%, respectively (9). Globally the prevalence of RE in children is increasing as well, partially due to the recent COVID epidemic and increase of eye load during online classes (10, 11).

The Lancet Global Health Commission (12) defines eye health as maximized vision, ocular health, and functional ability, essential to achieve many of the United Nations (UN) Sustainable Development Goals (SDGs). Undetected or uncorrected RE might cause a number of complications in school-aged children, especially in those who represent the vulnerable groups (4, 12). However, in many cases those adverse outcomes are avoidable and could be prevented by providing timely access to the eye care education in order to detect and correct RE (13). In this light, knowledge, attitude and practice (KAP) study might provide aid, as the structured survey method that allows to access the current situation regarding specific prophylaxis methods among target audience and explore barriers for better compliance.

Previously identified and discussed barriers for RE correction include the concerns about cosmetic appearance, cost of spectacles, and inadequate knowledge levels among children, their parents and teachers (13, 14). Some studies reported that older participants are more comfortable with wearing glasses (15), while lack of knowledge and fear of complications become barriers for refractive surgery (16). However, for younger children the main source of knowledge and attitude is their parents or guardians (17–19). At that, the knowledge among parents remain low in many countries, including China (20, 21). Moreover, parental migration is a widespread phenomenon in China and Europe, leading to the millions of children left in the care of grandparents and other guardians (22, 23). Parental migration is detrimental to the health of left-behind children and adolescents, including the evidence of lower correction rate with eyeglasses in myopia (24), but its effect on children’s education and self-care regarding RE is still barely discussed.

This study aimed to explore knowledge, attitudes and practice regarding RE management among left-behind children of migrant workers or their temporary guardians.

## 2 Methods

### 2.1 Study design and participants

This cross-sectional study was conducted by the Ophthalmology Department of Beijing Jishuitan Hospital between July and August, 2023, and included left-behind children of migrant workers from the mountainous areas of Hebei, Henan, Jiangxi, Liaoning and Zhejiang Province, China. Due to practical considerations it was not possible to perform eye examination of children during the questionnaire filling, so records of refractive test results from compulsory school examination in the spring of 2023 have been used. The dioptric results were computer refraction results obtained without any cycloplegic drug administration to the eyes. Inclusion criteria: (1) child in the care of guardian is aged 8–14 years; (2) parents have been migrant workers for more than 6 months; (3) child was tested for refractive status with the official medical record without cycloplegic drug administration; and (4) voluntarily participation in this survey. Guardians of children with organic eye diseases, or with incomplete school examination and eye refraction test results were excluded. The study has been ethically approved by the Ethics Committee of Beijing Jishuitan Hospital and informed consent has been obtained from all study participants.

The questionnaire design was referenced from “*Kanski’s Clinical Ophthalmology: A Systematic Approach, 9th ed*” (25), modified with comments from two chief physicians and professors and tested for reliability (150 copies) with Cronbach’s *α* = 0.835, suggesting good internal consistency. The final questionnaire was in Chinese (a version translated into English was attached as an Supplementary material Table) and included 4 dimensions of information collection with a total of 47 items. Of these, the basic information consisted of 10 items; the knowledge dimension consisted of 14 items, with correct responses scored as one and wrong or unclear responses scored as 0, with possible scores ranging from 0 to 14. The attitudes and practice dimensions consisted of 9 and 14 items respectively, both using a five-point Likert scale, with scores ranging from 9 to 45 and 14–70, with 5, 4, 3, 2 and 1 or 1, 2, 3, 4, 5 being assigned from very positive, positive, neutral, negative and very negative, respectively. In the Attitude dimension for questions A1, A4, A6, A9 replies “agree” and “strongly agree” were considered positive attitude, while for questions A2, A3, A5, A7, A8 replies “agree” and “strongly agree” were considered negative attitude and reverse score was used. The specific rules of each items in the practice dimensions were showed in the Appendix. “School eye exercises” referred to 1–5 min eye massage exercises under the teacher’s guidance, implemented in all Chinese schools to accelerate blood circulation, relax eye muscles and eliminate eye fatigue (26).

The children from mountainous areas such as Sanhe Area in Hebei, Shangqiu Area in Henan, Shangrao Area in Jiangxi, Chaoyang and Dalian Area in Liaoning, and Jinhua Area in Zhejiang were selected via convenience sampling; questionnaires were distributed in schools (guardians were contacted beforehand and asked to sign informed consent form). All 8 researchers had experience in conducting self-administered questionnaire surveys and underwent the relevant quality control training. All questionnaires were paper-based. If the child is unable to fill in the questionnaire effectively, the temporary guardian or the researchers will help complete the questionnaire. Once questionnaires have been collected, they were screened again by a member of the research team to eliminate incomplete questionnaires and to record data from complete questionnaires. Members of the researcher team checked all questionnaires for completeness, internal coherence and reasonableness.

### 2.2 Statistical analysis

The statistical analysis software was SPSS 26.0 (IBM Corp., Armonk, NY, USA). Continuous variables were described using Mean ± SD, and comparisons between groups were made using ANOVA. Categorical indicators were described using frequency (percentages). Pearson’s correlation analysis was used to explore correlations among KAP scores. Multivariate logistic regression analysis was applied for factors associated with KAP, using 70% of the score distribution as the cut off value. Univariate variables with *p* < 0.05 were used for multivariate analysis. Structural Equation Modeling (SEM) was used to explore the pathways between basic information, each KAP items and KAP scores. The SEM was based on the following hypothesis (H): H1: The knowledge had positive effect on attitude. H2: The attitude had positive effect on practice. H3: The knowledge had positive effect on practice. All tests were two-sided, with *p* < 0.05 indicating statistical significance. Two-sided *p*-values <0.05 were considered statistically significant.

## 3 Results

Initially, a total of 471 questionnaires were collected, after elimination of 121 unqualified responses due to incomplete data, a total of 350 (74.31%) valid questionnaires were included for analysis. The majority of guardians of the child were grandparents (93.14%), maternal grandparents (2.86%) or other relatives (4.00%). Among children, 27.14% lived away from parents for >5 years, 39.15% had myopia, 3.71% had hyperopia, and 11.71% had astigmatism. The mean knowledge, attitude and practice scores of participants were 9.21 ± 3.00 (possible range 0–14), 33.23 ± 3.57 (possible range 9–45) and 50.19 ± 5.31 (possible range 14–70), respectively (Table 1).

**Table 1:** Distribution of demographic characteristics and comparison of KAP scores among different subgroups of demographic characteristics.

Variables	*N* (%)	RE* prevalence	Knowledge, mean ± SD	*p*	Attitude, mean ± SD	*p*	Practice, mean ± SD	*p*
Total		39.14%	9.21 ± 3.00		33.23 ± 3.57		50.19 ± 5.31	
Gender				0.378		0.055		0.011
Male	186 (53.14)	39.78%	9.05 ± 3.12		32.95 ± 3.72		49.61 ± 5.28	
Female	164 (46.86)	38.41%	9.38 ± 2.85		33.56 ± 3.37		50.84 ± 5.29	
Age, years				<0.001		<0.001		0.011
8	37 (10.57)	21.62%	10.46 ± 1.46		32.81 ± 2.47		52.05 ± 3.72	
9	40 (11.43)	15.00%	11.25 ± 1.30		31.53 ± 1.77		50.78 ± 4.04	
10	53 (15.14)	39.62%	10.72 ± 1.54		31.74 ± 2.25		50.49 ± 4.41	
11	60 (17.14)	33.33%	9.07 ± 2.80		33.53 ± 4.07		49.35 ± 5.14	
12	81 (23.14)	46.91%	7.02 ± 3.61		33.46 ± 3.92		48.70 ± 5.99	
13	66 (18.86)	54.55%	8.94 ± 2.91		34.58 ± 3.93		50.52 ± 6.20	
14	13 (3.71)	61.54%	8.85 ± 2.38		36.23 ± 3.32		53.31 ± 4.70	
Grade				<0.001		<0.001		0.030
1–4	136 (38.86)	28.68%	10.76 ± 1.48		32.36 ± 2.79		50.99 ± 4.09	
5–7	214 (61.14)	45.79%	8.22 ± 3.28		33.79 ± 3.89		49.68 ± 5.91	
Body mass index, kg/m^3^				<0.001		0.053		0.128
<18	169 (48.29)	39.05%	9.03 ± 3.08		33.62 ± 3.54		49.90 ± 5.66	
18–24	159 (45.43)	38.99%	9.72 ± 2.74		32.84 ± 3.58		50.72 ± 4.83	
>24	22 (6.29)	40.91%	6.91 ± 2.99		33.09 ± 3.44		48.59 ± 5.61	
Residence				<0.001		<0.001		<0.001
Sanhe, Hebei	50 (14.29)	30.00%	11.76 ± 0.48		31.76 ± 0.77		53.56 ± 1.39	
Shangqiu, Henan	50 (14.29)	34.00%	7.12 ± 2.34		34.42 ± 2.87		45.44 ± 5.19	
Shangrao, Jiangxi	100 (28.57)	54.00%	6.44 ± 3.01		34.48 ± 4.51		50.31 ± 6.42	
Chaoyang, Liaoning	50 (14.29)	36.00%	10.56 ± 1.25		32.38 ± 3.30		48.30 ± 5.00	
Dalian, Liaoning	50 (14.29)	36.00%	11.2 ± 1.05		30.10 ± 1.17		50.98 ± 2.97	
Jinhua, Zhejiang	50 (14.29)	30.00%	10.94 ± 1.2		35.02 ± 2.73		52.42 ± 3.31	
Guardians				0.002		0.290		0.006
Grandparents	326 (93.14)	39.57%	9.32 ± 2.96		33.27 ± 3.53		50.30 ± 5.24	
Maternal grandparents	10 (2.86)	20.00%	8.90 ± 3.60		33.70 ± 3.53		52.40 ± 5.15	
Others (relatives, family friends, etc.)	14 (4.00)	42.86%	6.79 ± 2.36		32.14 ± 4.37		45.93 ± 5.46	
Duration away from parents (years)				0.003		0.015		0.318
<3	97 (27.71)	43.30%	9.21 ± 3.32		32.89 ± 3.27		49.78 ± 5.31	
3–5	158 (45.14)	29.11%	9.73 ± 2.53		32.91 ± 3.38		50.64 ± 5.16	
>5	95 (27.14)	51.58%	8.34 ± 3.18		34.14 ± 4.01		49.85 ± 5.55	
Myopia				0.086		0.084		0.001
No	213 (60.86)	–	9.39 ± 2.98		32.94 ± 3.35		50.83 ± 5.03	
Yes	137 (39.14)	–	8.92 ± 3.01		33.69 ± 3.85		49.20 ± 5.60	
Hyperopia				0.040		0.596		0.501
No	337 (96.29)	–	9.26 ± 2.98		33.23 ± 3.55		50.14 ± 5.33	
Yes	13 (3.71)	–	7.85 ± 3.18		33.46 ± 4.22		51.46 ± 4.82	
Astigmatism				0.725		0.530		0.519
No	309 (88.29)	–	9.25 ± 2.94		33.29 ± 3.54		50.19 ± 5.36	
Yes	41 (11.71)	–	8.88 ± 3.42		32.83 ± 3.80		50.17 ± 5.01	
Parents with RE				<0.001		0.461		0.018
Yes	95 (27.14)	60.00%	10.07 ± 2.68		33.21 ± 3.59		50.75 ± 4.32	
No	233 (66.57)	29.18%	9.10 ± 3.00		33.15 ± 3.53		50.31 ± 5.41	
Unclear	22 (6.29)	54.55%	6.59 ± 2.58		34.18 ± 3.91		46.50 ± 6.75	
Hours of sleep per day				0.003		0.236		0.001
7 h and below	11 (3.14)	63.64%	6.55 ± 3.27		33.09 ± 5.96		43.55 ± 5.92	
7 ~ 9 h	273 (78.00)	37.00%	9.46 ± 2.84		33.11 ± 3.43		50.20 ± 5.34	
More than 9 h	66 (18.86)	43.94%	8.62 ± 3.31		33.79 ± 3.63		51.24 ± 4.24	
Hours of reading and writing per day	2.80 ± 1.97	–	–	–	–	–	–	–
Hours of using electronic devices	1.56 ± 1.29	–	–	–	–	–	–	–

*RE: Refractive error, including myopia [equivalent spherical refraction (SER) ≤ −0.50 D], hyperopia (moderate-to-high: SER ≥ +2.00 D), and astigmatism [cylinder (CYL) ≥ 0.5D or ≤ −0.50D], based on records of compulsory school examination without cycloplegic drug administration; Knowledge score: 14 items, possible scores ranging from 0 to 14; Attitudes score: 9 items, five-point Likert scale, scores ranging from 9 to 45; Practice score: 14 items, five-point Likert scale, scores ranging from 14 to 70.

In the knowledge dimension the question with the highest rate of correct answers was “When child is taking an online course at home, the room should be well lit and the brightness of electronic equipment should be adjusted appropriately, not too bright or too dark” (correct rate of 89.71%). Questions that appeared to be more difficult, with the lowest rate of correct answers excluding control ones, were “Refractive errors include myopia, hyperopia and astigmatism” and “Child can inherit myopia from their parents” (correct rates of 61.14 and 66.57%, respectively) (Table 2).

**Table 2:** Distribution of knowledge scores regarding refractive error among left-behind children of migrant workers.

Items	*N* (%)	
	Wrong	Correct
K1 Refractive errors include myopia, hyperopia and astigmatism.	136 (38.86)	214 (61.14)
K2 The main manifestation of myopia is a lack of clarity in seeing at a distance.	92 (26.29)	258 (73.71)
K3 You can inherit myopia from your parents.	117 (33.43)	233 (66.57)
K4 Myopia of −4.00 or more is considered a severe myopia.	119 (34)	231 (66)
K5 Mild astigmatism may be slightly uncomfortable, but with severe astigmatism you may feel that you are seeing vague or doubled images, not clear from far and near.	100 (28.57)	250 (71.43)
K6 Inappropriate eye usage, such as regular eye rubbing, may aggravate astigmatism.	108 (30.86)	242 (69.14)
K7 To fully rest your eyes, you need to get up every 20 min while working and studying and stand in front of a window and look 20 feet (6 m) away for at least 20 s.	58 (16.57)	292 (83.43)
K8 When you are taking an online course at home, try to ensure that the room is well lit and that the brightness of your electronic equipment is adjusted appropriately, not too bright or too dark.	36 (10.29)	314 (89.71)
K9 Outdoor exercises are also crucial to myopia prevention and control, and with proper protective measures you should spend more than 2 h in outdoor activities every day.	39 (11.14)	311 (88.86)
K10 You should not read at home in too bright or too dark light, and if possible, ensure that the room lighting and the eye lamp are switched on at the same time.	45 (12.86)	305 (87.14)
K11 Wearing frame glasses is one of the proper ways to control myopia, while reducing the frequency of reading/studying at close range and increasing outdoor activities are also important.	86 (24.57)	264 (75.43)
K12 When your eyes become tired and dry, blink often to relieve the feeling.	85 (24.29)	265 (75.71)
K13 Diet and sleep also have an effect on the onset and progression of myopia.	92 (26.29)	258 (73.71)

In the attitude dimension 66.0% agreed and 20.57% strongly agreed to participate in the future promotion activities related to eye care for the sake of children in their care. Questions that provoked disagreement the most were designed this way, such as “it is unnecessary to have eyesight checked regularly” (56.86% disagreed and 22.57% strongly disagreed), “there is no effect of body position during reading or using electronic devices on eyesight” (59.43% disagreed and 32.0% strongly disagreed) and “RE are not a serious problem” (67.14% disagreed and 24.0% strongly disagreed). However, more than 1/3 of participants disagreed that it is necessary to wear glasses even if vision is not clear; 40.86% of participants expressed concerns related to the appearance if RE made it necessary to wear glasses, and 7.71% more reported possible self-esteem problems related to having RE (Table 3).

**Table 3:** Distribution of attitude scores regarding refractive error among left-behind children of migrant workers.

Items	*N* (%)				
	Strongly agree	Agree	Neutral	Disagree	Strongly disagree
A1 I think I should wear glasses as long as I cannot see clearly.	20 (5.71)	177 (50.57)	28 (8)	106 (30.29)	19 (5.43)
A2* I do not think it’s necessary to have eyesight checked regularly if I can see clearly.	2 (0.57)	58 (16.57)	12 (3.43)	199 (56.86)	79 (22.57)
A3* I do not think lying down when reading a book or playing with electronic devices has any effect on my eyesight.	3 (0.86)	21 (6)	6 (1.71)	208 (59.43)	112 (32)
A4 I would like to participate in promotion activities on eye care designed for primary and middle school students.	72 (20.57)	231 (66)	23 (6.57)	19 (5.43)	5 (1.43)
A5* Refractive error is not a serious disease and there is no need to pay attention to eye usage and protection in daily life.	2 (0.57)	16 (4.57)	13 (3.71)	235 (67.14)	84 (24)
A6 If I had refractive error, I will be concerned about its effects on my life, my studies and even my employment in the future.	16 (4.57)	128 (36.57)	20 (5.71)	160 (45.71)	26 (7.43)
A7* I would be really worried about my appearance if I need glasses to correct refractive error.	5 (1.43)	138 (39.43)	69 (19.71)	99 (28.29)	39 (11.14)
A8* If I had refractive error, it would lower my self-esteem.	1 (0.29)	27 (7.71)	46 (13.14)	204 (58.29)	72 (20.57)
A9 If the school, community or hospital organized a “vision protection” parent–child activities, I would like to participate.	90 (25.71)	221 (63.14)	28 (8)	5 (1.43)	6 (1.71)

*For questions A1, A4, A6, A9 replies “agree” and “strongly agree” were considered positive attitude, while for questions A2, A3, A5, A7, A8 replies “agree” and “strongly agree” were considered negative attitude and reverse score was used.

During last year 54.86% of responders participated in seminars or activities related to the eye health care, organized by schools; 91.14% of children performed eye massage exercises controlled by teacher at school, at least occasionally, and 92.0% had their eyesight checked at schools at least once a year. However, around 10% of participants reported to often use electronic devices in the dark or read books, watch mobile phone and other electronic devices while lying down. Finally, majority of participants acknowledged the necessity to visit hospital upon the signs of visual impairment in the child in their care (77.14%) and ensure a rest from reading to prevent tiredness of eyes (76.43%) (Table 4).

**Table 4:** Distribution of practice scores regarding refractive error among left-behind children of migrant workers, *n* (%).

Items	Yes	No			
P1 1. Does your school have multi-media teaching?	281 (80.29)	69 (19.71)			
P2 Has your school organized seminars or activities on eye care for children in the past year?	192 (54.86)	158 (45.14)			
	Always	Often	Occasionally	Rarely	Never
P3 Do you do eye exercises at school?	147 (42)	137 (39.14)	35 (10)	19 (5.43)	12 (3.43)
	Never	Average 1 ~ 2 times per week	Average 3 ~ 4 times per week	Average 5 times per week and above	
P4 How often do you have a vision checked at school or hospital each year?	28 (8)	151 (43.14)	85 (24.29)	86 (24.57)	–
P5 How much egg, meat, fish or animal liver do you consume each week?	34 (9.71)	190 (54.29)	92 (26.29)	34 (9.71)	–
P6 How much dairy or soy products do you consume each week?	11 (3.14)	106 (30.29)	129 (36.86)	104 (19.62)	–
P7 How much fresh fruit and vegetables do you consume each week?	1 (0.29)	64 (18.29)	110 (31.43)	175 (50)	–
	Totally accordance	Accordance	Not sure	Discordance	Totally discordance
P8 When reading and writing, do like this: keep your eyes one foot away from the table; Chest one punch away from the book; Hold the pen one inch away from the tip of the pen.	29 (8.29)	67 (19.14)	179 (51.14)	63 (18)	12 (3.43)
P9 I have the habit of watching TV, mobile phones and tablet computer in the dark.	6 (1.71)	33 (9.43)	71 (20.29)	176 (50.29)	64 (18.29)
P10 I have the habit of lying down while reading books, watching mobile phone and other electronic devices.	6 (1.71)	38 (10.86)	55 (15.71)	188 (53.71)	63 (18)
P11 I have the habit of reading books or watching electronic devices on mobile transport (busses, cars).	5 (1.43)	13 (3.71)	48 (13.71)	204 (58.29)	80 (22.86)
P12 I have the habit of rubbing my eyes.	40 (11.43)	74 (21.14)	65 (18.57)	147 (42)	24 (6.86)
P13 If I cannot see clearly, I will go to the hospital for a formal examination rather than going to an optician to get a pair of glasses.	163 (46.57)	107 (30.57)	41 (11.71)	21 (6)	18 (5.14)
P14 If I feel my eyes are tired, I will take a distant view, go outdoors, or close my eyes to rest.	177 (50.57)	94 (26.86)	50 (14.29)	17 (4.86)	12 (3.43)

**Figure 1:**
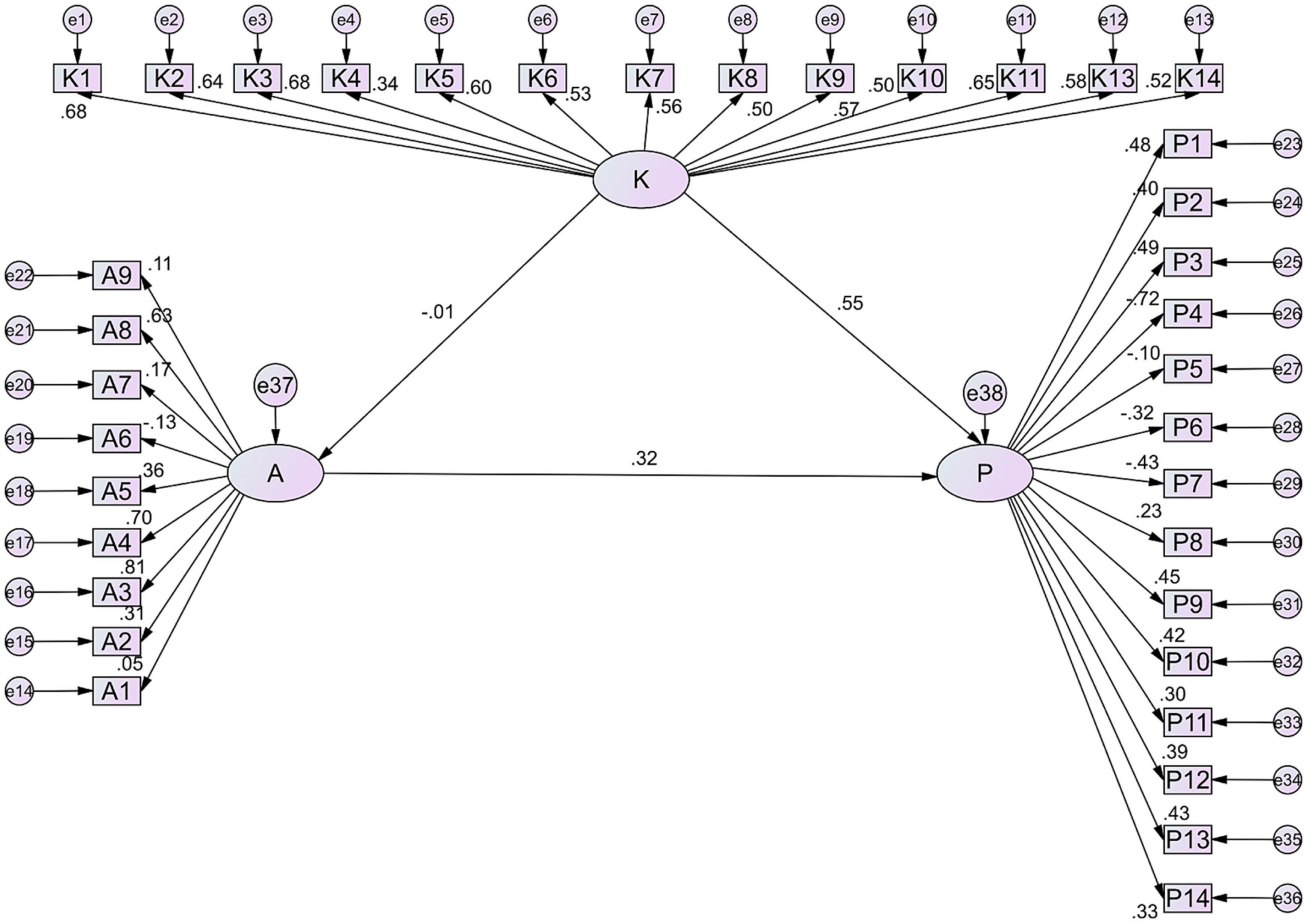
The structural equation model. The structural equation model is a comprehensive statistical approach to testing hypotheses about relations among observed (Knowledge, Attitude, and Practice) and latent variables (the questions for each dimension, such as K1 ~ K14). (1) Graphic shape: The latent variables are typically represented by ellipses or circles, observed variables are generally depicted as rectangles or squares, and error terms are illustrated by small circles connected to the corresponding observed variables. (2) Arrows: The arrows and coefficients among the three KAP constructs indicate both the direction and strength of the relationships between the latent variables. The small circles associated with A and P represent the residual terms. A unidirectional arrow signifies the direction of causation, specifically from cause to effect. When an arrow points from one latent variable to another, it indicates that the former exerts a direct effect on the latter. Conversely, if the latent variable is directed from the observed variable, it signifies that the latent variable is represented by that observed variable. (3) Number: The numerical value on the arrow connecting the latent variable to the observed variable signifies the factor loading, which reflects the strength of the association between the observed variable and the latent variable, specifically the regression coefficient of the observed variable with respect to the latent variable. A positive value indicates a positive correlation, suggesting that as the latent variable increases, the observed variable also tends to increase; conversely, a negative value indicates a negative correlation, implying that as the latent variable increases, the observed variable tends to decrease. The figure was created by AMOS software.

Pearson’s correlation analysis showed positive correlation was found between knowledge and practice (*r* = 0.286, *p* < 0.001), negative correlation between knowledge and attitude (*r* = −0.150, *p* = 0.005), and positive correlation between attitude and practice (*r* = 0.141, *p* = 0.008). Multivariate logistic regression analysis showed that age of children (OR = 0.748, 95%CI: 0.632–0.885, *p* = 0.001), duration of life without parents >5 years (OR = 0.345, 95%CI: 0.172–0.691, *p* = 0.003), and RE in parents (OR = 0.405, 95%CI: 0.218–0.753, *p* = 0.004) were independently associated with knowledge (Table 5). Children’s age, grade, residence and duration away from parents was associated with attitude score (all *p* < 0.05, Table 6). Children’s gender (female, OR = 1.671, 95%CI: 1.006–2.777, *p* = 0.047), relation to the child other than grandparent (OR = 0.252, 95%CI: 0.064–0.999, *p* = 0.050), and duration of children’s sleep per day (OR = 8.401, 95%CI: 1.473–47.923, *p* = 0.017) were independently associated with practice (Table 7). In addition, the SEM analysis confirmed that the impact of knowledge on attitude was negative, but without statistical significance (*β* = −0.03, *p* > 0.05), while impact of knowledge on practice was significantly positive (*β* = 1.251, *p* < 0.001) (Figure 1 and Table 8).

**Table 5:** Multivariate logistic regression analysis of factors associated with the better knowledge of refractive error among left-behind children of migrant workers.

Variables	Univariate logistic regression analysis		Multivariate logistic regression analysis	
	OR (95%CI)	*p*	OR (95%CI)	*p*
Gender				
Male	REF*			
Female	1.026 (0.669–1.573)	0.907		
Age, years	0.713 (0.621–0.819)	<0.001	0.748 (0.632–0.885)	0.001
BMI				
<18	REF		REF	
18–24	1.65 (1.051–2.589)	0.03	1.827 (1.082–3.087)	0.024
>24	0.229 (0.081–0.65)	0.006	0.272 (0.086–0.86)	0.027
Guardians				
Grandparents	REF		REF	
Maternal grandparents	0.945 (0.262–3.415)	0.931	1.305 (0.284–5.998)	0.732
Others (relatives, friends, etc.)	0.105 (0.023–0.477)	0.004	0.284 (0.052–1.555)	0.147
Duration away from parents (years)				
<3	REF		REF	
3–5	0.966 (0.567–1.645)	0.898	0.708 (0.37–1.356)	0.298
>5	0.391 (0.218–0.702)	0.002	0.345 (0.172–0.691)	0.003
Myopia				
No	REF		REF	
Yes	0.627 (0.405–0.97)	0.036	0.695 (0.399–1.211)	0.199
Hyperopia				
No	REF			
Yes	0.573 (0.188–1.742)	0.326		
Astigmatism				
No	REF			
Yes	0.959 (0.495–1.858)	0.901		
Parents with RE				
Yes	REF		REF	
No	0.51 (0.301–0.862)	0.012	0.405 (0.218–0.753)	0.004
Unclear				
Average hours of sleep per day				
7 h and below	REF		REF	
7 ~ 9 h	7.909 (1.675–37.335)	0.009	4.667 (0.728–29.922)	0.104
More than 9 h	4.235 (0.85–21.113)	0.078	3.092 (0.46–20.769)	0.245

*REF indicates a value used as a reference in the comparison.

**Table 6:** Univariate logistic regression analysis of factors associated with more positive attitude toward refractive error among left-behind children of migrant workers.

Variables	Univariate logistic regression analysis	
	OR (95%CI)	*p*
Knowledge	1.03 (0.955–1.111)	0.442
Gender		
Male	REF*	
Female	1.452 (0.913-2.311)	0.115
Age, years		
BMI	1.128 (0.985–1.29)	0.081
<18	REF	
18–24	0.537 (0.331–0.87)	0.012
>24	0.525 (0.205–1.343)	0.179
Guardians		
Grandparents	REF	
Maternal grandparents	1.645 (0.343–7.89)	0.534
Others (relatives, friends, etc.)	0.411 (0.14–1.204)	0.105
Duration away from parents (years)		
<3	REF	
3–5	0.632 (0.361–1.107)	0.109
>5	1.087 (0.565–2.09)	0.803
Myopia		
No	REF	
Yes	1.104 (0.688–1.769)	0.682
Hyperopia		
No	REF	
Yes	1.427 (0.384–5.293)	0.595
Astigmatism		
No	REF	
Yes	0.701 (0.355–1.386)	0.307
Parents with RE		
Yes	REF	
No	1.128 (0.676–1.882)	0.645
Unclear	3.068 (0.844–11.155)	0.089
Average hours of sleep per day		
7 h and below	REF	
7 ~ 9 h	1.201 (0.343–4.212)	0.774
More than 9 h	2.571 (0.648–10.206)	0.179

*REF indicates a value used as a reference in the comparison.

**Table 7:** Multivariate logistic regression analysis of factors associated with better practice regarding refractive error among left-behind children of migrant workers.

Variables	Univariate logistic regression analysis		Multivariate logistic regression analysis	
	OR (95%CI)	*p*	OR (95%CI)	*p*
Knowledge	1.215 (1.126–1.312)	<0.001	1.204 (1.1–1.318)	<0.001
Attitude	1.064 (0.997–1.135)	0.060		
Gender				
Male	REF*		REF	
Female	1.818 (1.152–2.867)	0.010	1.671 (1.006–2.777)	0.047
Age, years	0.846 (0.739–0.969)	0.016	1.021 (0.861–1.21)	0.813
BMI				
<18	REF		REF	
18–24	0.968 (0.608–1.541)	0.889	0.906 (0.532–1.543)	0.717
>24	0.381 (0.155–0.936)	0.035	0.585 (0.208–1.646)	0.309
Guardians				
Grandparents	REF		REF	
Maternal grandparents	1.848 (0.386–8.854)	0.443	1.961 (0.366–10.496)	0.432
Others (relatives, friends, etc.)	0.126 (0.034–0.461)	0.002	0.252 (0.064–0.999)	0.050
Duration away from parents (years)				
<3	REF			
3–5	1.314 (0.766–2.255)	0.322		
>5	0.884 (0.491–1.592)	0.682		
Myopia				
No	REF		REF	
Yes	0.411 (0.26–0.649)	<0.001	0.398 (0.238–0.665)	<0.001
Hyperopia				
No	REF			
Yes	1.682 (0.454–6.231)	0.437		
Astigmatism				
No	REF			
Yes	0.748 (0.382–1.463)	0.396		
Parents with RE				
Yes	REF			
No	0.847 (0.504–1.422)	0.529		
Unclear	0.418 (0.162–1.075)	0.070		
Average hours of sleep per day				
7 h and below	REF		REF	
7 ~ 9 h	8.853 (1.874–41.82)	0.006	3.511 (0.669–18.421)	0.138
More than 9 h	15.3 (2.978–78.619)	0.001	8.401 (1.473–47.923)	0.017

*REF indicates a value used as a reference in the comparison.

**Table 8:** Structural equation modeling of relationship between knowledge, attitude and practice toward refractive error (RE) in left-behind children of migrant workers.

		SE	*p*
Knowledge on attitude	−0.0003	0.012	0.831
Attitude on practice	4.004	4.515	0.375
Knowledge on practice	1.251	0.201	<0.001

## 4 Discussion

This study demonstrated that left-behind children of migrant workers might have sufficient knowledge, positive attitude and proactive practice toward RE management, which were possibly influenced by child’s age and gender, RE in parents and duration of living without parents, as well as the duration of children’s sleep per day. These results are specific for the population and might be used to correct the course of eye care education programs currently in use.

Due to the recent rise in the ocular disease burden worldwide, correction of the RE attracts significant scientific interest (27). This study assesses one of the vulnerable populations, such as young children of migrant workers who were left in care of temporary guardians, but the resulting KAP scores in children were not significantly lower than previously reported for parents. In particular, in the study by Assefa et al. (14) the overall proportion of high school children who had good knowledge and a favorable attitude toward RE was 53.8 and 52.1%, respectively. Other studies in school children or young adults demonstrated comparable or lower scores (28–30). In this study, around 10% of participants reported unacceptable practice of eye care. Promising results are at least partly explained by the strong school educational and practical programs – more than half of responders participated in seminars or activities related to the eye health care, organized by schools, and more than 90% occasionally performed eye exercises at school. Rate of eyesight checks in the schools was also higher compared to some other reports (16, 20), but still 8% of children under the care of temporary guardians never had their eyesight checked. Although procedure is obligatory in schools, those results draw special attention to the fact that a notable proportion of children might miss it, most likely due to the recent COVID epidemic and online schooling.

Unfavorable attitude of parents or guardians toward RE correction can have long-term consequences in children, leading to the loss of educational opportunities, loss of economic gain in the future and impaired quality of life (14, 15). In previous reports around 1/3 of responders were against wearing spectacles due to esthetic reasons or believes that wearing could damage their eyes (14, 19, 21). In line with that, 1/3 responders in this study answered that they do not believe in necessity of wearing glasses even if vision is not clear, citing concerns related to the appearance and self-esteem. Interesting to note that knowledge scores were getting lower as the child in responders’ care get older, with the most notable difference between grade one-four and grade five-seven students. Those observations contrast with the previous studies, where knowledge was getting better depending on age, with university students demonstrating more responsibility in eye care (14, 16). It might be at least partly explained by the natural transfer of responsibility from the guardian to child, as the child is getting older and more independent. There is another probability that children of older age experienced longer separation from their parents – knowledge scale scores in this study were also lower with the longer duration of separation. Attitude scores were increasing gradually with child’s age and duration of separation from parents, demonstrating the increasing responsibility in self-care, while practice scores were distributed sporadically. Moreover, the weak inverse correlation was found between knowledge and attitude, but was not confirmed by SEM, suggesting that attitude scores might be influenced by other factors rather than knowledge; guardians getting older as children growing, and duration of separation from working parents are likely to be among those factors. Therefore observed changes might be peculiar for the study population and should be taken into account during the future educational interventions.

It is important to note that this study found that children who get to sleep <7 h per day had considerably lower knowledge and practice scores, compared to those who sleep more. Overall proportion of participants with poor sleep quality among children was only 3.14%, which is not higher compared to the population of children living with parents in China; but with the older age this number could potentially became much higher (31, 32). In the previous study undertaken among Chinese school students, incidence of myopia increased with the decrease in sleep duration (7). In addition to authors discussing various influencing factors, such as oxidative stress and longer usage of electronic devises in children with insomnia, eye hygiene and eye care might also contribute to the development of myopia and other RE cases.

According to previous surveys, majority of school-year children obtain their information regarding eye-care from family rather than teachers (15, 18). With parents away due to the labor migration, the temporary guardians need to fulfill the role of reliable information source. Although it was shown before that knowledge and practice regarding RE was lacking in Chinese parents (19, 21), majority of participants in this study demonstrated acceptable results. However, in the small sub-population of children whose guardians other than grandparents, KAP scores were disturbingly low, suggesting that this group is the most vulnerable; relationship to the child other than grandparents was independently associated with lower practice scores. In addition, many children in the absence of parental guidance look for supplement information on the internet, such as Tiktok videos. Recent study by Ming et al. (33) discussed that myopia-related content on Tiktok should be treated with caution, pointing out moderate-to-poor reliability and variable quality across video sources; providing access to the comprehensive and accurate information should be, therefore, a priority in planning educational programs.

This study has some limitations. The sampling was limited by the need to obtain the information of the “migrant worker” status of parents from schools, and originally planned sample size was not reached; furthermore, the number of questionnaires distributed in each area was decided by research assistants, which might have led to selection bias. In addition, the target group was left-behind children from remote and economically disadvantaged regions, also, most of their guardians were older adult, and thus the initial idea to use combination of paper and electronic questionnaires to broaden sampling returned poor results. This study was focused on left-behind children, however, the actual presence of RE in children has not been confirmed by cycloplegic refraction. While sufficient KAP are essential for the RE prevention regardless current status, we discussed distribution of RE based on the school examination reports, thus there is a possibility that reported numbers might not reflect the overall population. Finally, social expectations bias is applicable for the majority of questions, as the responders might have chosen answers they perceive to be “right.”

## 5 Conclusion

In conclusion, left-behind children of migrant workers might have mostly sufficient knowledge, positive attitude and proactive practice toward RE management, which were possibly influenced by child’s age and gender, RE in parents and duration of living without parents, as well as duration of children’s sleep per day.

## Data Availability

The original contributions presented in the study are included in the article/supplementary material, further inquiries can be directed to the corresponding author.
