# Supplementary material for: Knowledge, attitudes and practice toward refractive errors management among left-behind children of migrant workers
Source: Front Public Health. 2025 Jan 21;12:1373209. doi: 10.3389/fpubh.2024.1373209 (PMC11790664; doi:10.3389/fpubh.2024.1373209)
Supplement: Supplementary file 1 [file Table_1.docx]

| Dear Child/Guardian,  Hello!  We are healthcare professionals from XX Hospital conducting a study on eye habits and refractive errors. We need to collect some information to investigate the knowledge, attitudes, and practices of Chinese children aged 8-14 and their guardians regarding eye habits and refractive status. Our aim is to help more children like yours improve their vision in the future.  If you and your child participate in this study, we will need you to do two things:   1. Have your child's eyes undergo a refractive examination, including tests for vision, refractive error, and eye axis length. 2. Complete this questionnaire based on your actual situation.   Although participating in this study will take up some of your time, please rest assured that it will not cause you any harm. All information will be kept confidential and your data will not be disclosed. We sincerely appreciate your taking the time out of your busy schedule to support our scientific research!  Thank you very much!  □I have been informed and agreed to the use of the collected data for scientific research. |
| --- |

| **Part I Basic Information** | |
| --- | --- |
| 1.Child's gender: | a.Male  b.Female |
| 2.Child's age: , grade: . | |
| 3.Child's height: , weight: . | |
| 4.Your residence: . | |
| 5.Your guardian is: | a.Grandparents  b.Maternal grandparents  c.Other (relatives, friends, etc.)  d.No one to look after me |
| 6.Duration of away from parents: months per year, for a total of years. | |
| 7. Do you have myopia, hyperopia or astigmatism? | a.Yes  b.No  c.Unclear |
| 7.1If yes, the degrees are, myopia: left eye____degrees, right eye____degrees; hyperopia: left eye____degrees, right eye____degrees; astigmatism: left eye____degrees, right eye____degrees. | |
| 8. Was your father or mother myopic? | a.Yes  b.No  c.Unclear |
| 9.Average hours of sleep per day: h. | |
| 10.Average time spent reading and writing per day: h, Average time spent using electronic devices: h. | |

| **Part II Knowledge of eye habits and refractive error** | |
| --- | --- |
| 1.Refractive errors include myopia, hyperopia and astigmatism.  a.Correct; b.Wrong; c.Unclear |  |
| 2.The main manifestation of myopia is a lack of clarity in seeing at a distance.  K3 You can inherit myopia from your parents.  a.Correct; b.Wrong; c.Unclear |  |
| 1. Myopia is inherited. 2. a.Correct; b.Wrong; c.Unclear |  |
| 4.Myopia of -4.00 or more is considered a severe myopia.  a.Correct; b.Wrong; c.Unclear |  |
| 5.Mild astigmatism may be slightly uncomfortable, but with severe astigmatism you may feel that you are seeing vague or doubled images, not clear from far and near.  a.Correct; b.Wrong; c.Unclear |  |
| 6.Inappropriate eye usage, such as regular eye rubbing, may aggravate astigmatism.  a.Correct; b.Wrong; c.Unclear |  |
| 7.To fully rest your eyes, you need to get up every 20 minutes while working and studying and stand in front of a window and look 20 feet (6m) away for at least 20 seconds.  a.Correct; b.Wrong; c.Unclear |  |
| 8.When you are taking an online course at home, try to ensure that the room is well lit and that the brightness of your electronic equipment is adjusted appropriately, not too bright or too dark.  a.Correct; b.Wrong; c.Unclear |  |
| 9.Outdoor exercises are also crucial to myopia prevention and control, and with proper protective measures you should spend more than 2 hours in outdoor activities every day.  a.Correct; b.Wrong; c.Unclear |  |
| 10.You should not read at home in too bright or too dark light, and if possible, ensure that the room lighting and the eye lamp are switched on at the same time.  a.Correct; b.Wrong; c.Unclear |  |
| 11.Wearing frame glasses is one of the proper ways to control myopia, while reducing the frequency of reading/studying at close range and increasing outdoor activities are also important.  a.Correct; b.Wrong; c.Unclear |  |
| 12.Wearing glasses regularly can aggravate myopia and make it more and more intense.  a.Correct; b.Wrong; c.Unclear |  |
| 13. When your eyes become tired and dry, blink often to relieve the feeling.  a.Correct b.Wrong; c.Unclear |  |
| 14. Diet and sleep also have an effect on the onset and progression of myopia.  a.Correct; b.Wrong; c.Unclear |  |

| **Part III Attitude to eye habits and refractive error** |
| --- |
| 1. I think I should wear glasses as long as I can't see clearly. (P)  a.Strongly agree; b.Agree; c.Neutral; d.Disagree; e.Strongly disagree |
| 2. I don't think it's necessary to have eyesight checked regularly if I can see clearly. (N)  a.Strongly agree; b.Agree; c.Neutral; d.Disagree; e.Strongly disagree |
| 3. I don't think lying down when reading a book or playing with electronic devices has any effect on my eyesight. (N)  a.Strongly agree; b.Agree; c.Neutral; d.Disagree; e.Strongly disagree |
| 4. I would like to participate in promotion activities on eye care designed for primary and middle school students. (P)  a.Strongly agree; b.Agree; c.Neutral; d.Disagree; e.Strongly disagree |
| 5. Refractive error is not a serious disease and there is no need to pay attention to eye usage and protection in daily life. (N)  a.Strongly agree; b.Agree; c.Neutral; d.Disagree; e.Strongly disagree |
| 6. If I had refractive error, I will be concerned about its effects on my life, my studies and even my employment in the future. (P)  a.Strongly agree; b.Agree; c.Neutral; d.Disagree; e.Strongly disagree |
| 7. I would be really worried about my appearance if I need glasses to correct refractive error. (N)  a.Strongly agree; b.Agree; c.Neutral; d.Disagree; e.Strongly disagree |
| 8. If I had refractive error, it would lower my self-esteem. (N)  a.Strongly agree; b.Agree; c.Neutral; d.Disagree; e.Strongly disagree |
| 9. If the school, community or hospital organized a "vision protection" parent-child activities, I would like to participate. (P)  a.Strongly agree; b.Agree; c.Neutral; d.Disagree; e.Strongly disagree |

| **Part IV Practice on eye habits and refractive error** |
| --- |
| 1. Does your school have multi-media teaching? (a=5, b=1)   a.Yes; b.No |
| 2. Do you do eye exercises at school? (a~e=5~1)  a.Always; b.Often; c.Occasionally; d.Rarely; e.Never |
| 3. Has your school organized seminars or activities on eye care for children in the past year? (a=5, b=1)  a.Yes; b.No |
| 4. How often do you have a vision checked at school or hospital each year? (a~d=2~5)  a.Never; b.Once a year; c.Twice a year; d.More than twice a year |
| 5. How much egg, meat, fish or animal liver do you consume each week? (a~d=2~5)  a.Never; b.Average 1~2 times per week; c.Average 3~4 times per week; d.Average 5 times per week and above |
| 1. How much dairy or soy products do you consume each week? (a~d=2~5)   a.Never; b.Average 1~2 times per week; c.Average 3~4 times per week; d.Average 5 times per week and above |
| 1. How much fresh fruit and vegetables do you consume each week? (a~d=2~5)   a.Never; b.Average 1~2 times per week; c.Average 3~4 times per week; d.Average 5 times per week and above |
| 1. When reading and writing, do like this: keep your eyes one foot away from the table; Chest one punch away from the book; Hold the pen one inch away from the tip of the pen. (a~e=5~1)   a.Totally accordance; b.Accordance; c.Not sure; d.Discordance; e.Totally discordance |
| 1. I have the habit of watching TV, mobile phones and tablet computer in the dark. (a~e=1~5)   a.Totally accordance; b.Accordance; c.Not sure; d.Discordance; e.Totally discordance |
| 1. I have the habit of lying down while reading books, watching mobile phone and other electronic devices. (a~e=1~5)   a.Totally accordance; b.Accordance; c.Not sure; d.Discordance; e.Totally discordance |
| 1. I have the habit of reading books or watching electronic devices on mobile transport (buses, cars). (a~e=1~5)   a.Totally accordance; b.Accordance; c.Not sure; d.Discordance; e.Totally discordance |
| 1. I have the habit of rubbing my eyes. (a~e=1~5)   a.Totally accordance; b.Accordance; c.Not sure; d.Discordance; e.Totally discordance |
| 1. If I can't see clearly, I will go to the hospital for a formal examination rather than going to an optician to get a pair of glasses. (a~e=5~1)   a.Totally accordance; b.Accordance; c.Not sure; d.Discordance; e.Totally discordance |
| 1. If I feel my eyes are tired, I will take a distant view, go outdoors, or close my eyes to rest. (a~e=5~1)   a.Totally accordance; b.Accordance; c.Not sure; d.Discordance; e.Totally discordance |
